# Economic and environmental impacts of a resource-saving committee in a Japanese hemodialysis clinic: a case study

**DOI:** 10.3389/frhs.2025.1737266

**Published:** 2026-01-15

**Authors:** Kei Nagai, Hiroshi Kajiyama, Tadaatsu Hoshino, Sho Hata, Keisuke Nansai, Rei Kawashima, Hideo Kawashima

**Affiliations:** 1 Department of Nephrology, Hitachi General Hospital, Ibaraki, Japan; 2 Department of Nephrology, Faculty of Medicine, University of Tsukuba, Ibaraki, Japan; 3 Syu Jin Kai, Kawashima Clinic, Hitachi, Japan; 4 Material Cycles Division, National Institute for Environmental Studies, Ibaraki, Japan

**Keywords:** cost-saving, electricity, hemodialysis, medical waste, water consumption

## Abstract

Dialysis therapy is a resource-intensive treatment for end-stage kidney disease that remains highly dependent on in-center hemodialysis in Japan. From both economic and environmental perspectives, it is necessary to reduce energy consumption and resource use, and minimize waste generation to achieve sustainable kidney healthcare. The clinic targeted in this study provides hemodialysis in a regional city and launched a resource-saving committee in 2008 to implement initiatives, appoint green champions, and monitor four environmental items (electricity, gas and water consumption, and waste generation) and financial effects. To retrospectively evaluate environmental impact, we calculated the carbon footprint. The median monthly consumption of electricity, gas, and water per hemodialysis patient was approximately 353 kWh, 17 m^3^, and 9 m^3^, respectively. These levels of resource consumption were nearly equivalent to those of an average Japanese household in 2022. Switching to a combination of city water and well water reduced both costs and environmental impact. However, the overall financial benefit and initial investment burden, such as for installation of light-emitting diode fixtures and developing the water supply system, were not fully investigated. The resource-saving committee appears to have mitigated both economic and environmental impacts to some extent; however, steady resource-saving efforts were accompanied by surging costs of electricity and medical waste disposal during the study period, indicative of recent general inflation in Japan. To achieve more sustainable dialysis therapy that balances environmental and health considerations, further proactive initiatives are needed to reduce resource use beyond the current scope, such as through individualized dialysate prescriptions.

## 1 Introduction

The number of patients with chronic kidney disease is growing worldwide (1) as well as in Japan (2). Dialysis therapy is essential for patients with end-stage kidney disease, and global demand is expected to increase (3). Hemodialysis is a resource-intensive healthcare, especially at the point of care (4). In France and Australia, the amounts of electricity and water consumed, and the waste generated per session have been estimated as approximately 7–16 kWh, 370–380 L, and 1.1 kg, respectively (5, 6). Therefore, the economic costs associated with providing this therapy must be considered to ensure the sustainability of dialysis services (7).

The concept of “Green Dialysis”, which addresses environmental concerns related to dialysis therapy that consumes large amounts of resources, has gained widespread acceptance (8, 9). The metric that converts greenhouse gases (the cause of global warming) into CO_2_ equivalents is termed the carbon footprint (CFP). The CFP of healthcare has shown a generally increasing trend, accounting for approximately 4.6% of annual global carbon emissions in 2011 and 6.1% in 2019 (10, 11). Healthcare is thus emerging as one of the most carbon-intensive service sectors and a major contributor to climate change (12). The annual CFP per patient receiving dialysis therapy has been estimated as 3.9 tCO_2_e, compared with 0.31 tCO_2_e in individuals without dialysis (13). Efforts are also underway to mitigate the environmental impact of dialysis treatment by reducing resource consumption. For example, in France, monthly data collection (eco-reporting) was implemented in hemodialysis centers in 2005, successfully reducing power and water consumption by 29.6% (from 23.1 to 16.26 kWh/session) and 52% (from 801 to 382 L/session), respectively (5). Care-related waste decreased from 1.8 to 1.1 kg as a result of regular staff training. Across a cohort of 2,642 patients, an estimated 102,440 tCO_2_e of carbon savings were achieved, equivalent to the CO_2_ emissions produced by a plane flying around the globe 11,500 times (5). These findings demonstrate that visualizing and monitoring resource consumption in dialysis can drive practice improvements and promote behavioral change among staff, leading to resource conservation.

The clinic in this study provides in-center hemodialysis therapy, but not home dialysis, peritoneal dialysis, or transplantation, in a regional city. Since initiating hemodialysis services in 1978, the clinic has maintained financially stable operations primarily through dialysis practices, implementing economical resource-saving measures across all departments. We aimed to achieve Green Dialysis by assessing sequential changes in resource consumption from both in economic and environmental perspectives and identifying effective strategies to reduce quantitative burdens, in the first such initiative in Japan.

## 2 Methods

### 2.1 Concept and establishment of a resource-saving committee in a hemodialysis facility

With the aim of achieving sustainable dialysis therapy, we established a resource-saving committee in the clinic in 2008 (Kawashima Clinic, Hitachi, Japan). The committee aims to enhance medical efficiency and quality through the conservation of organizational resources. Its membership comprises representatives from among Physicians, Nursing Department, Caregiver Department, Laboratory Department, Nutrition Department, and Administrative Department. The committee strives to operate at a scale appropriate to its size and departmental composition. Furthermore, we aimed to clarify the roles of the resource-saving committee members and have them become leaders of sustainability initiatives as “Green Champions” within their respective departments.

We prioritized resource-saving efforts for utilities (electricity, gas, and water), which constitute a significant portion of the operational expenses. As these savings involve lifestyle habits, changing them requires a shift in staff mindset. To thoroughly implement the initiative, we conducted monthly all-staff meetings to report on and display the results of the conservation activities. We discussed proposals from each department within the committee and worked to enhance the savings efforts by staff. In response to rising electricity and water bills, the facility planned and introduced energy-efficient light emitting diode fixtures and a groundwater pumping and treatment system, starting in 2012. We maintained the following key principles for resource savings in healthcare: the predominant consideration is that healthcare quality must not be compromised, and necessary expenses for patients and users should be met without hesitation.

### 2.2 Checklist used for implementing changes

In 2008, the committee compiled a checklist of 25 items including lighting, water usage, supplies, paper towels, elevators, air conditioning, toilet paper, meals, patient waiting rooms, computers, and office equipment (Supplementary Table S1). Using the checklist, committee members investigated and tracked current usage levels, identified necessary vs. unnecessary items, and confirmed implementation plans for resource savings with each department. This initiative was strictly limited to daily self-improvement efforts by dialysis staff and did not involve any changes to dialysis prescriptions that could affect patient health outcomes (such as switching from hemodialysis to hemodiafiltration, or altering dialysate volume or temperature), or any changes to outpatient care methods. Therefore, the initiatives had no direct influence on patient outcomes.

### 2.3 Monitoring of resources at the point of care in a hemodialysis facility

The resource-saving committee recorded the usage amounts (kWh and m^3^) and fees (yen) for electricity, gas, and water from the respective companies' invoices. For waste disposal, the fees for medical waste and general waste processing were available, but there was no information on their weight or volume. The findings of this investigation summarized the total annual costs for the entire facility from the beginning of 2016 to the end of 2024. The activities of the resource-saving committee were consistent and proactive during the nine-year period under investigation, and the number of patients remained largely stable, ranging from 237 to 275.

### 2.4 Assessment of carbon footprint

A CFP, direct and indirect emissions of greenhouse gases (GHG), for the items monitored were calculated with a tiered-hybrid life cycle assessment approach (14) where the CFP for each item (electricity, city gas, and water consumption, and waste treatment cost) is estimated by multiplying the quantity of that item by its embodied GHG emission intensity derived by an input-output model. We obtained the emission intensities of each item *e_i_* for the relevant year from the 3EID (Embodied Energy Emission Intensity Data) database (15) that provides those of Japanese commodity sectors.

$$CFP_{i}=e_{i}y_{i}$$

Here, *y_i_* represents the quantity consumed of item *i*, and *e_i_* denotes the GHG emission intensity per unit consumption of item *i*.

### 2.5 Statistical analysis

To examine the significance of changes in resource costs and CFP over time, we divided the data into three periods (2016–2018, 2019–2021, and 2022–2024). Since the sampled values are three per period, it is evident that the data are not normally distributed; therefore, we described the median and interquartile range (IQR). Statistical significance of the differences among median values was evaluated using the Mann–Whitney *U*-test as a nonparametric test (GraphPad Prism version 7, GraphPad Software, San Diego, CA).

### 2.6 Ethics

The present study collected no personal data from patients or healthcare providers, or expose either group to any risk. Hence, formal ethics approval was not required.

## 3 Results

### 3.1 Reduction in resource consumption in in-center hemodialysis therapy through sustainability initiatives

**Figure 1:**
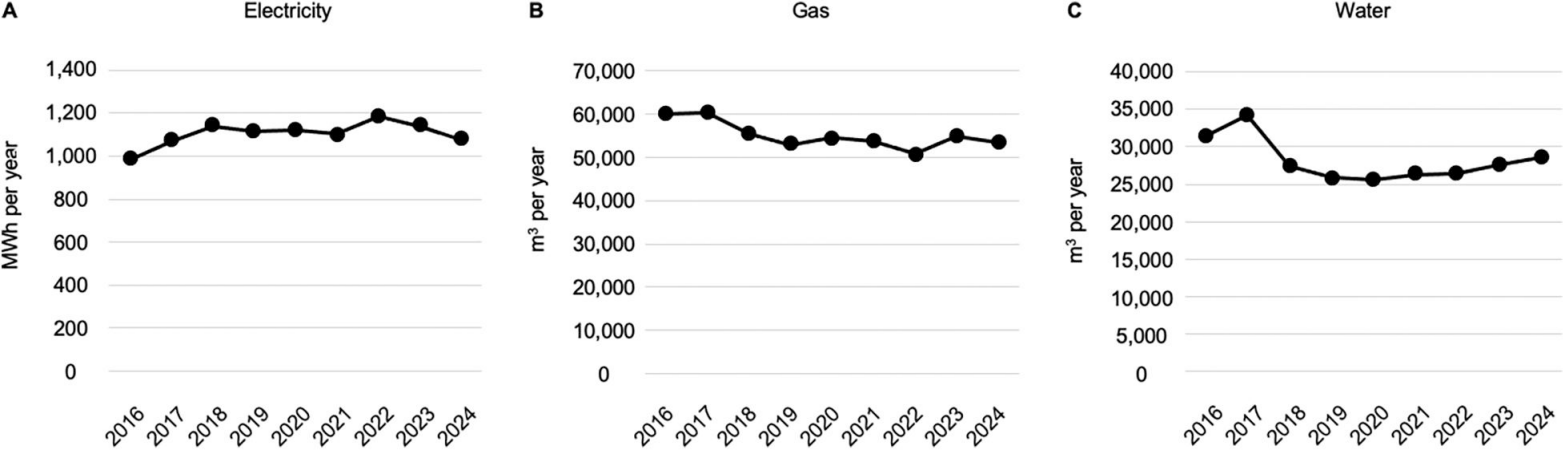
Annual amount of resource consumption at the facility in this study. Line graphs show the annual amounts of electricity **(A)**, gas **(B)**, and water **(C)** consumed at the hemodialysis facility for years 2016–2024.

The number of maintenance hemodialysis patients at the clinic fluctuated between a minimum of 237 (2016) and a maximum of 275 (2018) at year-end. Annual person-months also fluctuated between a minimum of 2,883 person-months (2016) and a maximum of 3,236 person-months (2022) (Supplementary Table S2). Figures 1A–C shows trends in electricity, gas, and water consumption. Through the ongoing activities of the resource-saving committee, annual consumption remained largely unchanged at approximately 1.0–1.2 million kWh of electricity, 50,000–60,000 m^3^ of gas, and 25,000–34,000 m^3^ of water. Because the number of dialysis patients fluctuates even within a single year, we converted these consumption figures to person-months as follows: monthly electricity consumption, 340–370 (median 353 and IQR 349–357) kWh per capita; gas consumption, 16–21 (median 17 and IQR 17–17) m^3^ per capita; and water consumption, 8–11 (median 9 and IQR 8–9) m^3^ per capita (Supplementary Table S2). In this study, we were unable to determine resource consumption per session because of data resolution.

### 3.2 Rising monetary burden for electricity and medical waste in current hemodialysis therapy

**Figure 2:**
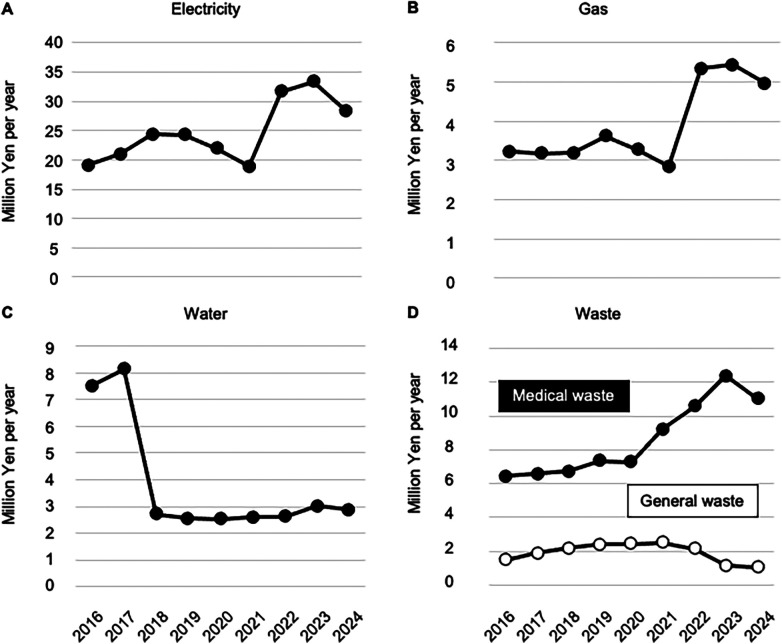
Economic cost of resources and waste disposal at the point of hemodialysis care. Line graphs show the total cost of electricity consumption **(A)**, gas use **(B)**, water use **(C)** and waste disposal **(D)** for the entire hemodialysis facility for years 2016–2024.

Energy resources were greatly affected by rising prices. Annual electricity costs rose from ¥19 million (Japanese Yen) in 2016 to a peak of ¥33 million in 2023 (Figure 2A), whereas annual gas costs rose from ¥3.2 million in 2016 to a peak of ¥5.4 million in 2024 (Figure 2B). To address rising water rates, a groundwater system was introduced in 2012. Since then, we have used both purchased city water and groundwater as source water for dialysis solutions, gradually increasing the proportion of groundwater to nearly 90%. Accordingly, annual water use costs fell from ¥8.1 million (2017) to ¥2.8 million (2024), depending on the price per unit (Figure 2C). While the annual cost for general waste disposal remained stable between ¥1.1 million and ¥2.4 million, that for medical waste disposal doubled from ¥6.4 million in 2016 to ¥12.4 million in 2023 (Figure 2D).

**Figure 3:**
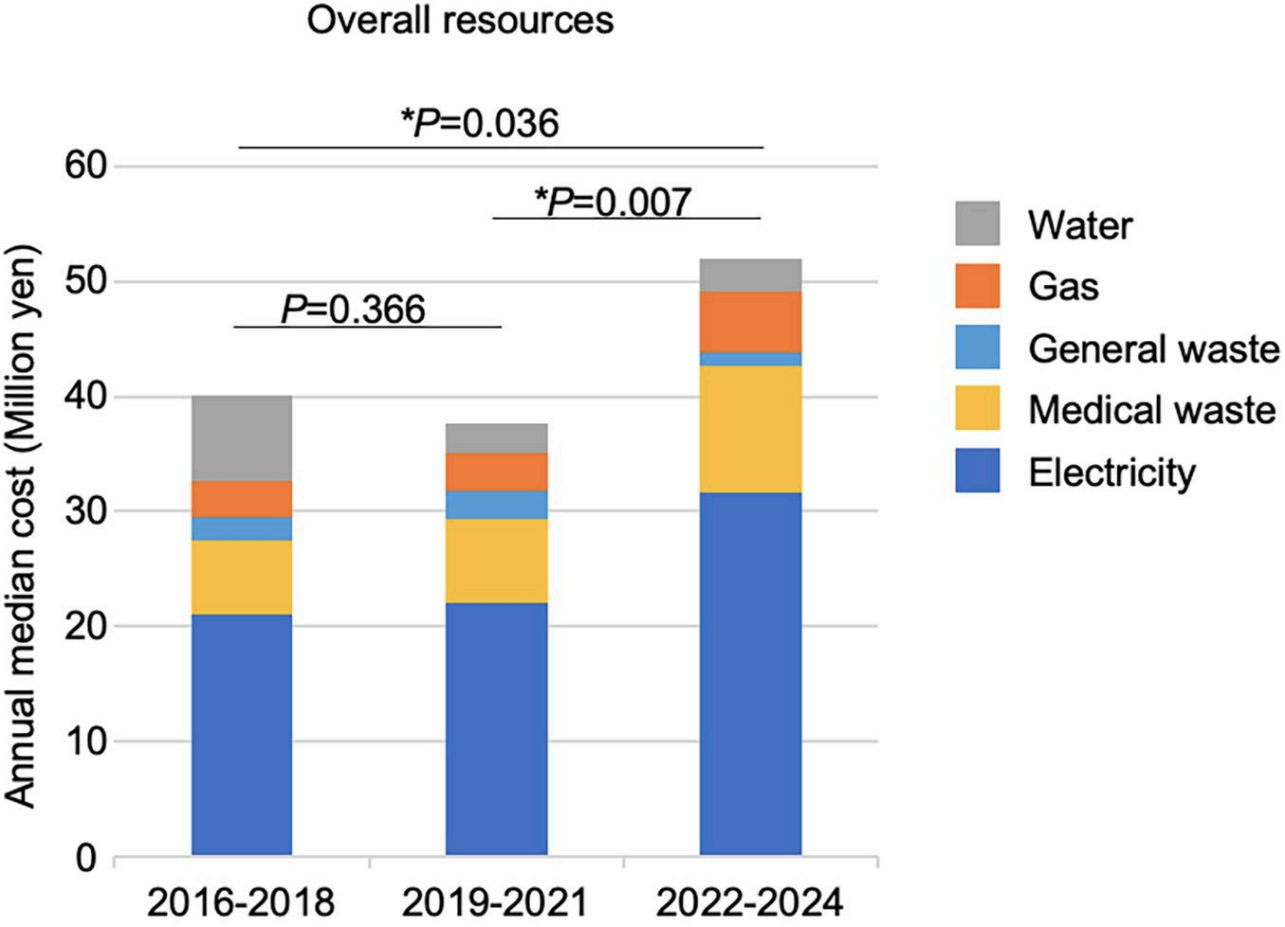
Temporal comparison of electricity, gas, and water consumption and medical waste costs at the facility in this study. Annual costs of resource consumption and waste disposal are broken down for each of the periods 2016–2018, 2019–2021, and 2022–2024.

By dividing the three periods (2016–2018, 2019–2021, and 2022–2024), a breakdown of costs is shown in Figure 3. The difference in total cost was not significant between 2016 and 2018 and 2019–2021. However, the meidan annual costs for electricity, gas, and medical waste disposal in 2022–2024 (¥31 million, ¥5.3 million, and ¥11.0 million, respectively) were significantly higher than those in the first period (¥21 million, ¥3.2 million, and ¥6.6 million, respectively).

### 3.3 Impact assessment of hemodialysis therapy on climate change

**Figure 4:**
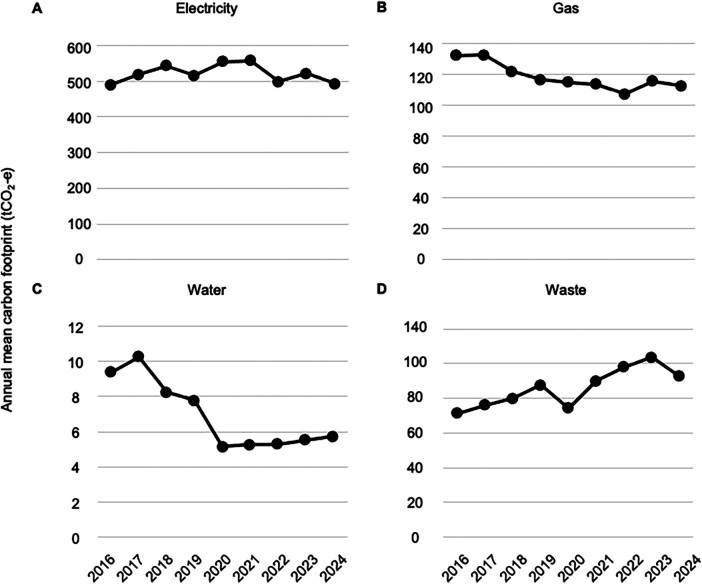
Estimated annual carbon footprint of resource consumption and waste at the facility in this study. Line graphs show the CFP of electricity **(A)**, gas **(B)**, and water consumption **(C)** as well as waste disposal **(D)** for the entire hemodialysis facility for years 2016–2024.

Greenhouse gases include several atmospheric gases responsible for global warming, primarily carbon dioxide and methane. The greenhouse gas emissions associated with hemodialysis can be calculated as the CFP using standardized publicly available conversion units. As a result of energy-saving measured implemented in the dialysis room, the annual electricity-derived CFP remained mostly unchanged at approximately 490–560 tCO_2_-e, which was substantially higher than that of gas (110–130 tCO_2_-e), water (5–10 tCO_2_-e), and waste disposal (70–100 tCO_2_-e) (Figures 4A–D). The overall CFP, consisting of electricity, gas, water, and waste, did not differ significantly among the three periods: 2016–2018 (annual median 736 tCO_2_-e and IQR 713–751 tCO_2_-e), 2019–2021 (annual median 762 tCO_2_-e and IQR 735–766 tCO_2_-e), and 2022–2024 (annual median 715 tCO_2_-e and IQR 706–731 tCO_2_-e) (Supplementary Figure S1). The breakdown data shows significant decrease in CFP of gas between 2016 and 2018 (annual median 132 tCO_2_-e and IQR 127–133 tCO_2_-e) and 2022–2024 (annual median 113 tCO_2_-e and IQR 110–114 tCO_2_-e, *P* = 0.02), and CFP of water use between 2016 and 2018 (annual median 9 tCO_2_-e and IQR 9–10 tCO_2_-e) and 2022–2024 (annual median 6 tCO_2_-e, min 5 tCO_2_-e and IQR 5–6 tCO_2_-e, *P* = 0.02), increase in CFP of waste disposal between 2016 and 2018 (annual median 76 tCO_2_-e and IQR 74–78 tCO_2_-e) and 2022–2024 (annual median 98 tCO_2_-e and IQR 95–101 tCO_2_-e, *P* = 0.01) (Supplementary Figure S1).

## 4 Discussion

Medical institutions need to pursue profit for business sustainability; thus, the activities of the resource-saving committee primarily aim to reduce economic burden. In recent years, addressing environmental issues has gained increasing attention. From a corporate social responsibility (CSR) perspective, conservation and energy efficiency have also become critical initiatives for healthcare professionals. The facility in this study did not increase overall resource consumptions and environmental impact during the period from 2016 to 2024. The CFP for electricity consumption has remained unchanged, while the CFP for gas and water consumption has decreased over time. However, it is possible that these achievements have been offset by the significant increase in the environmental cost of waste disposal or the impact of additional amount of infectious waste due to COVID-19. To rigorously prove causation, it would be preferable to compare the facility with another that did not implement resource-saving measures during the same period, or to compare with survey results from before the facility's initiatives began. However, due to limitations in the information collected for this survey, this could not be examined.

Our investigation revealed the median consumption of electricity, gas, and water per hemodialysis patient: monthly electricity consumption was approximately 353 kWh, gas consumption was 17 m^3^, and water consumption was 9 m^3^. For comparison, the monthly average resource consumption per average household in Japan is as follows: 329 kWh of electricity, 16 m^3^ of gas, and 9 m^3^ of water per capita (16, 17). Put simply, we are constantly consuming one additional set of household resources daily to sustain the life of a patient with end-stage kidney disease: a fact that healthcare providers involved in dialysis therapy must recognize. In a society where resources are secure, dialysis care can be provided, so this reality remains hidden. However, disasters are an exception. The clinic in this study was affected by the 2011 Great East Japan Earthquake and has experience providing dialysis during major disasters. As part of business continuity plan measures, reducing energy consumption during emergencies is essential because medical facilities have the predominant mission of maintaining operations even during disasters to serve the community and protect lives. We consider it is critically important to practice conservation and energy efficiency on a daily basis to sustain medical facilities when relying on limited emergency self-generated power sources.

From an economical perspective, although all staff members have worked together and succeeded in implementing daily cost-saving measures, the reality is that expenses remain strained by soaring utility costs and rising prices. The main reasons for the surge in purchased electricity costs are rising international fuel prices, depreciation of the yen, increased surcharges for renewable energy adoption, and tight electricity supply and demand. Efforts to curb rising utility costs within the healthcare sector are considered difficult. Despite requiring some initial investment, measures such as improving the energy efficiency of medical equipment, implementing on-site power generation (18), conserving gas and water through heat recovery systems, and reducing unnecessary disinfection water usage in dialysis equipment will become increasingly necessary. These measures will also likely become new priorities for the healthcare industry. It is also necessary for the dialysis clinics to make the management decision to introduce energy-saving and low-environmental-impact initiatives. In addition to publishing this paper, it is believed that information sharing among physicians through study groups and the expansion of subsidy programs for introducing equipment will be necessary.

Research examining the sustainability of dialysis therapy from an environmental perspective has increased significantly in recent years. However, the lack of internationally comparable data stems from the absence of appropriate environmental inventories in healthcare. In the present analysis, it was relatively easy to calculate the carbon footprints for electricity, gas, and water services because the corresponding unit costs for the relevant time period were publicly available. In contrast, the carbon footprint for medical waste disposal is still unavailable in Japan. We can refer to a pioneering case study regarding environmental assessment for healthcare, conducted in England (19). In that study, the CFP per unit of hospital waste disposal varied because different base units were assigned depending on the treatment process, and none matched the current review. For example, infectious waste generates 338 kg CO_2_e/ton from autoclave decontamination, 167 kg CO_2_e/ton from low temperature incineration, and 64 kg CO_2_e/ton from transportation, totaling 569 kgCO_2_e/ton of waste. This clearly demonstrates that infectious waste accounts for a much higher CFP than general waste, which is typically incinerated or landfilled. The ideal method to reduce the weight of medical waste in hemodialysis therapy is to recycle dialysis membranes and circuits, but this is not currently practical due to economic costs, infection risks, and ethical considerations (20). Therefore, efforts by industry, such as reducing the weight of dialysis membrane housings and redesigning circuits, are also required.

Although not discussed at meetings of the resource-saving committee in the clinic, advanced facilities are aiming to reduce environmental impact by individualizing and optimizing dialysate volume. In Japan as well, reducing the dialysate flow rate (Qd) to 400 ml/min during hemofiltration dialysis according to the patient's physical condition and residual renal function has been adopted in clinical practice (21). A blood flow rate (Qb) to Qd ratio of approximately 1:2 is considered efficient, and the Qd is occasionally adjusted accordingly in our experience. Regarding optimization of Qd, a single-center, nonrandomized, open-label, cross-over pilot trial involving 30 hemodialysis patients in India demonstrated a water conservation strategy by increasing dialysate temperature while reducing Qd without compromising the adequacy and safety of dialysis in young and hemodynamically stable patients (22). In that study, reducing the Qd from 500 ml/min to 300 ml/min achieved a 40% reduction in water consumption (22). Recently, discussions have begun and intensified within Japanese dialysis-related academic societies regarding the individual optimization of Qd in hemodiafiltration. We need to pay attention to this as an attempt to reduce the total volume of dialysate used indiscriminately, without causing adverse health effects.

Furthermore, if these resource-saving efforts extend to healthcare providers and to patients' households, they will make an important contribution to healthcare sustainability. From an environmental perspective, reducing wasteful consumption also allows us to play a vital role under any circumstances. In 2023, Japan conducted its first environmental awareness survey for dialysis facilities, the “Green Survey” (23). The results revealed that environmental awareness was not still high among Japanese dialysis staff, and few have implemented environmental actions. In four-fifths of the facilities (*n* = 208/255, 81.6%), no “green team” or resource-saving committee had been formed to promote environmental protection. As more environmentally conscious healthcare professionals emerge, it is hoped that patient-optimized dialysis care can be achieved through cooperation and understanding among physicians, healthcare staff, patients, and dialysis-related companies.

## Data Availability

The original contributions presented in the study are included in the article/Supplementary Material, further inquiries can be directed to the corresponding author.
