# Supplementary figures and images for: Economic and environmental impacts of a resource-saving committee in a Japanese hemodialysis clinic: a case study
Source: Front Health Serv. 2026 Jan 15;5:1737266. doi: 10.3389/frhs.2025.1737266 (PMC12852348; doi:10.3389/frhs.2025.1737266)

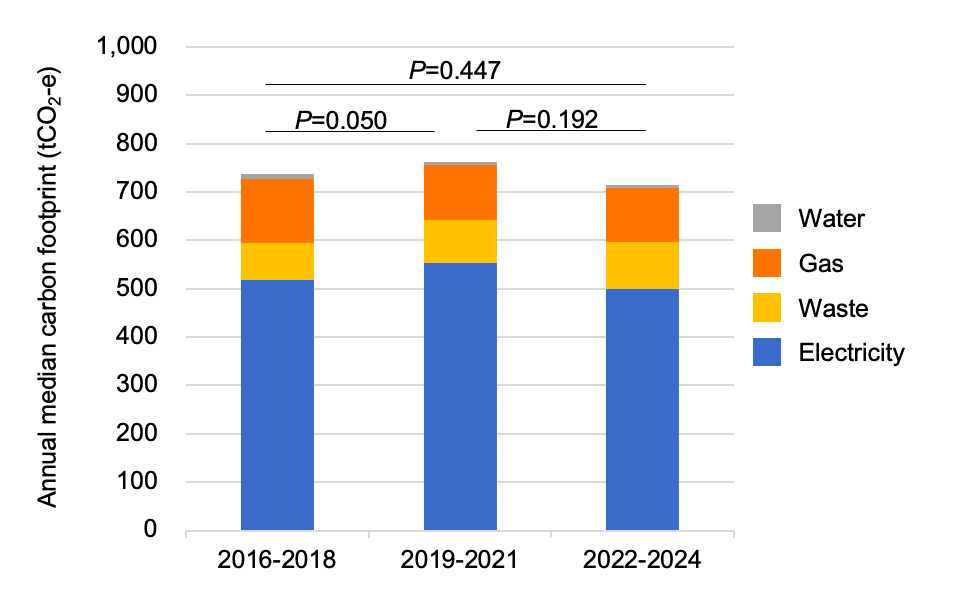

Supplement: Supplementary file 3 [file Image1.jpeg]
